# Anti-N-Methyl-D-Aspartate Receptor Encephalitis (ANMDARE) in a Patient With Hashimoto’s Thyroiditis

**DOI:** 10.7759/cureus.23109

**Published:** 2022-03-12

**Authors:** Aakangsha Jain, Jamie Thomas, Hernando Chong

**Affiliations:** 1 Osteopathic Medicine, Nova Southeastern University Dr. Kiran C. Patel College of Osteopathic Medicine, Fort Lauderdale, USA; 2 Medicine, Nova Southeastern University Dr. Kiran C. Patel College of Osteopathic Medicine, Fort Lauderdale, USA; 3 Family Medicine, Baptist Health South Florida, Miami, USA

**Keywords:** neurology and critical care, hashimoto’s encephalopathy, ovarian teratoma, hashimoto’s thyroiditis, anti nmda receptor encephalitis

## Abstract

Although encephalitis is more commonly caused by various infections, other etiologies that may rarely cause encephalitis must garner the attention of medical practitioners. In the realm of immune-mediated etiologies, anti-N-methyl-D-aspartate receptor encephalitis (ANMDARE) is the most common. It usually presents in a typical fashion with psychiatric symptoms followed by abnormal movements such as orofacial-lingual dyskinesia, tremor, dystonia, bradykinesia, ballism, or choreoathetosis occurring at or within the first month of onset, often affecting women and having a high correlation with ovarian teratomas. Our case report describes a 59-year-old Hispanic male with Hashimoto thyroiditis who presented with rapid cognitive decline. The diagnosis was confirmed with positive detection of NMDA receptor antibodies in the patient’s cerebrospinal fluid following a lumbar puncture. The patient was treated with the first-line therapy of intravenous (IV) immunoglobulins and corticosteroids with temporary relief of symptoms. Due to the rare occurrence and possible atypical presentation of ANMDARE, this case illustrates the importance of maintaining a high index of clinical suspicion when diagnosing a patient with an unknown cause of cognitive dysfunction, especially when considering various differentials based on the patient’s history.

## Introduction

Anti-N-methyl-D-aspartate receptor encephalitis (ANMDARE) is a well-defined autoimmune disorder. N-methyl-D-aspartate (NMDA) is an acidic amino acid that depolarizes neurons by selectively interacting with a distinct class of excitatory receptors. These receptors bind L-glutamate or structurally similar compounds [1]. They are distributed densely in the hippocampus, cerebral cortex, basal ganglia, and thalamus, as well as the brainstem and spinal cord [1]. 

In ANMDARE, self-reactive autoantibodies are formed against NR1 subunits of NMDA glutamate receptors [2-3]. Although ANMDARE is found in both men and women of all age groups, it is most commonly found in young female adults and has a strong association with ovarian teratomas [2]. Initial symptoms and presentation vary for different patient groups; however, most patients manifest symptoms within a similar spectrum. These include movement disorders, such as orofacial-lingual dyskinesias, tremor, dystonia, bradykinesia, ballism, or choreoathetosis [4]. Additional symptoms include autonomic dysregulation, central hypoventilation, seizures, memory deficits, and psychiatric disorders [5]. Psychiatric disorders, including behavioral disorders, psychosis, mood disorders, catatonia, and sleep disturbances, are the most common symptoms in N-methyl-D-aspartate receptor (NMDA), which frequently misleads physicians to a primary psychiatric diagnosis [3]. 

In patients with Hashimoto’s thyroiditis, Hashimoto’s encephalopathy (HE) is a potential complication. It is a clinically heterogeneous condition characterized by acute or subacute onset of neurological and/or psychiatric symptoms, associated with high titers of anti-thyroid peroxidase and anti-thyroglobulin antibodies [5]. ANMDARE and HE share similar clinical features, so the differential diagnosis can be difficult if specific antibodies are not tested. Misdiagnosis has negative implications for both the treatment and management of patients. Indeed, HE is typically steroid-responsive with favorable outcomes in the majority of cases, whereas intravenous immunoglobulin (IVIG), plasma exchange, and corticosteroids are the first-line treatments recommended for ANMDARE [6].

Here we present a unique case of ANMDARE in a male with a history of Hashimoto thyroiditis with no typical symptoms of seizures or psychiatric disturbances. This case iterates the importance of a high index of suspicion when approaching a patient with rapid cognitive decline and the importance of considering other encephalopathies as possible differentials. 

## Case presentation

Here we present a case of a 59-year-old Hispanic male on 10/05/2020 with a history of hypothyroidism, hyperlipidemia, atopic dermatitis, vitamin D deficiency, and erectile dysfunction who presented to his primary care physician accompanied by his wife due to difficulty concentrating. As per his wife, she noticed the patient had been behaving strangely for six months, acting more withdrawn and passive. After she received a phone call from his employers that he was having difficulty ambulating, she brought him to the office for further evaluation. The patient himself admitted he has been somewhat forgetful but denied any symptoms to suggest depression. He stated he is “eating regularly but unable to put on weight”. He denied any alcohol, tobacco, or illicit drug use. He was compliant with his medications which included Synthroid 150 mcg, Viagra 100 mg, and Vitamin D2 50,000 units. CBC, CMP, and urinalysis were all within normal limits. The lipid panel showed a LDL of 118 mg/dL (reference range < 100 mg/dL) and thyroid function study showed a TSH of 0.07 mIU/L (reference range 0.40 - 4.50 mIU/L). All other lab values were within normal limits. The patient’s Synthroid was lowered from 150 mcg to 100 mcg due to the low TSH level. 

Upon examination, the patient’s initial vital signs were within normal limits. The patient was well-nourished and well-developed and in no acute distress. On neurological exam, there were no focal neurological deficits, and gait was non-antalgic. On mental status exam, the patient was alert and oriented x 2 to time and place. He failed the three-word recall, failed serial sevens, spelled “world” backward after two attempts, and had a poor clock drawing with all the numbers drawn in the right upper quadrant. He had a flat affect but was cooperative with the examination. Before the office visit, a non-contrast MRI of the brain was ordered, which revealed mild brain atrophy with juxtacortical white matter signal changes compatible with chronic small vessel ischemia. Per the radiology report, the MRI showed no acute intracranial abnormalities. Due to the patient’s poor performance on the mini-mental status exam, the patient was immediately referred to the emergency department for further evaluation.

**Figure 1:**
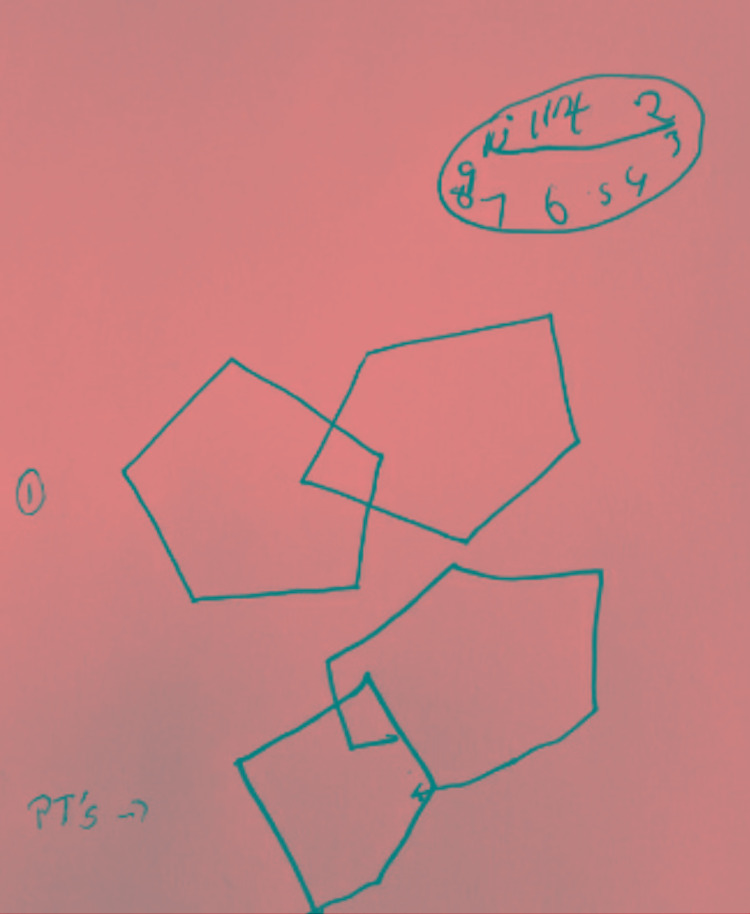
Bedside clock and hexagon drawing.

The patient was evaluated in the emergency department (ED) on the same day by the inpatient Neurology service, who recommended admission for further MRI studies with contrast and to consider a lumbar puncture (LP) due to what appeared to be rapid-onset dementia. Neurological examination revealed unremarkable motor and sensory findings. Bedside clock drawing was somewhat scribbled, and reproducing overlapping hexagons were crudely drawn (Figure 1).

According to the inpatient radiologist, repeat brain MRI without contrast revealed thin, diffuse, dural enhancement, which is nonspecific but seen with spontaneous intracranial hypotension. There were small bilateral subcortical cerebral T2 white matter hyperintensities which, according to the inpatient radiologist, are nonspecific but associated with chronic migraine headaches. Due to physical exam findings, unremarkable labs, and radiographic findings, the patient was diagnosed with rapidly progressive dementia with unknown etiology. Differentials at this time included viral encephalitis, autoimmune encephalitis, paraneoplastic syndrome, and prion disease. Inpatient neurology recommended a repeat contrast MRI of the brain, electroencephalography (EEG), lumbar puncture (LP), urine drug screen as well as anti-nuclear antibodies (ANA), HIV, thyroid panel, erythrocyte sedimentation rate, c-reactive protein, paraneoplastic panel, and NMDA in serum. 

On 10/08/2020, a lumbar puncture diagnosis of noninfectious was made due to results revealing CSF with no color, clear appearance, 0 white blood cells, 0 red blood cells, and absent xanthochromia.” ANA was positive with a titer of 1:40 (abnormal), consistent with a possible autoimmune cause of presentation. The patient was advised to remain inpatient to begin treatment with IV steroids and IV immunoglobulins (IVIG) due to possible autoimmune etiology. He was discharged on 10/12/2020 upon stabilization of symptoms and completion of steroid and IVIG treatment. On 10/13/2020, the results of the autoimmune panel returned, revealing a cerebrospinal fluid (CSF) NMDA titer of 1-80 and a negative serum NMDA titer. Results were relayed to the primary outpatient Neurologist who was following the patient.

On 11/14/2020, the patient was readmitted to the ED due to a relapse of encephalopathy symptoms. According to the patient’s wife, the patient had continued to have abnormal behavior with a depressed state and confusion before his readmission. In the ED, physical exam findings were normal; however, the patient was found to have a distant affect. Laboratory findings revealed positive anti-thyroid peroxidase antibodies and antithyroglobulin antibodies, which raised suspicion for steroid-responsive encephalopathy or Hashimoto encephalitis. The patient was also evaluated by inpatient hematology-oncology for a possible neoplastic syndrome; however, positron emission tomography (PET) scan results were negative. The patient was started again on IV methylprednisolone for five days with the improvement of symptoms. The patient was discharged with 60 mg of Prednisone daily to be tapered over several months, which would be closely followed by his primary Neurologist. 

Over the next several months, the patient was regularly following up with his primary care physician and neurologist and has been compliant with his tapering dose of prednisone but has continued to have cognitive and physical decline. The patient’s most recent PET scan from 07/12/2021 revealed decreased fluorodeoxyglucose (FDG) metabolism throughout the left cerebral hemisphere and the contralateral right cerebellum, consistent with crossed cerebellar diaschisis. It was also noted that there was subtle relative hypometabolism in the high right parietal lobe and right temporal lobe. Although he has been routinely attending physical therapy and maintaining his medication regimen, he has continued to have a progressive decline in cognition, balance, and fine motor skills. 

## Discussion

Albeit being a rare and potentially fatal syndrome, anti-N-methyl-D-aspartate receptor encephalitis (ANMDARE) is the most common immune-mediated encephalitis. It is mediated by autoantibodies against the NR1 subunit of the NMDA receptor [2-3]. ANMDARE is found to occur in both genders at any age, but most commonly in young adult women with ovarian teratomas [2]. In a retrospective study of 501 patients with ANMDARE, 81% were female, and 38% had an underlying neoplasm [7]. Our patient presented with a unique case due to being a male with no underlying paraneoplastic syndrome found on lab results or a PET scan. 

Additionally, patients with ANMDARE typically develop initial psychiatric symptoms followed by abnormal movements. During the first month of the disease, the six characteristic clinical manifestations include behavioral disturbances or cognitive dysfunction, speech disorders, epileptic seizures, movement disorders, compromise of the level of consciousness, autonomic symptoms, and hypoventilation, with 90% of patients showing at least four of these [8]. Our patient demonstrated an atypical initial presentation due to only having two of the six characteristic manifestations, cognitive dysfunction, and motor disorders - these are two of the factors required for diagnosis [3]. 

Fluorodeoxyglucose-positron emission tomography (FDG-PET) has been found to have a high sensitivity for the most severe forms of ANMDARE [7]. For this patient, it is remarkable that no initial workups, including EEG, serum, MRI, or PET scan, showed any pathologic feature regarding NMDARE. The first sign of movement disorder presented after six months; however, it was not evident on a PET scan until seven months following diagnosis. Repeat PET scan at that time revealed decreased FDG metabolism throughout the left cerebral hemisphere and the contralateral right cerebellum, consistent with crossed cerebellar diaschisis. It was also noted that there was subtle relative hypometabolism in the high right parietal lobe and right temporal lobe. Additionally, the patient had no NMDA receptor antibodies present in serum but had positive NMDA receptor antibodies in CSF, which confirmed his diagnosis. In a retrospective case-control study involving 250 patients with ANMDARE, 100% of the patients had NMDA receptor antibodies in their CSF, but only 86% of the patients had them in their serum, suggesting antibody testing of the CSF is more sensitive than using serum [7].

The patient in our case study also presented with concurrent Hashimoto thyroiditis. Hashimoto’s encephalopathy (HE) is a potential rare complication in patients with Hashimoto’s thyroiditis, with only 121 reported cases according to a systematic review done in 2006. On laboratory results, our patient was found to have elevated anti-thyroid peroxidase and anti-thyroglobulin antibodies. This finding, along with the acute or subacute onset of neurological or psychiatric symptoms, is characteristic of HE. The clinical presentation is frequently more insidious, with the pathognomic feature of HE being altered mental status, which presents as confusion with or without psychosis, seizures, myoclonus, or loss of consciousness [9-10]. There are two patterns of cognitive dysfunction present in HE: an acute to subacute stroke-like pattern and a more progressive, gradual pattern resulting in dementia, hallucination, or coma [9]. ANMDARE and HE share similar clinical features, so differential diagnosis can be difficult if specific antibodies are not tested. Although our patient had anti-thyroid peroxidase antibodies and anti-thyroglobulin antibodies, HE is a diagnosis of exclusion; therefore, with the identification of anti-NMDA receptor antibodies in this patient’s CSF, the diagnosis of ANMDARE superseded that of HE. 

Misdiagnosis has negative implications for both treatment and management of patients with ANMDARE and HE. Indeed, HE is typically steroid-responsive with favorable outcomes in the majority of cases, whereas IVIG, plasma exchange, and corticosteroids are the first-line treatments recommended for ANMDARE [6]. Most patients with ANMDARE respond well to first-line immunotherapy and the removal of a teratoma. In patients without a tumor or with delayed diagnosis, additional treatment with second-line immunotherapy (cyclophosphamide or rituximab) is usually needed [3]. Although they have not been systematically verified in large cohorts, possible third-line options include tocilizumab, methotrexate, bortezomib, azathioprine, and mycophenolate mofetil [7]. In general, symptoms may persist for weeks to months and then gradually recover with or without sequelae. Early detection and treatment are crucial for morbidity-free recovery; however, since recurrence is common, physicians need to be vigilant throughout the patient’s life. Our patient’s symptoms initially resolved during both ED visits following administration of IV methylprednisolone; however, since discharge, the patient has continued to progressively decline even with continuous prednisone treatment.

## Conclusions

Current studies suggest that ANMDARE may be the most common encephalitis; however, prevalence rates are difficult to estimate based on the rarity and difficulty in diagnosis of this disease. Due to its vague presentation coupled with the delayed onset of neurologic symptoms, physicians need to maintain a high index of suspicion. Here we present a unique case of a patient with Hashimoto thyroiditis diagnosed with ANMDARE who presented with rapid mental cognitive decline. His atypical presentation made the diagnosis challenging as various etiologies of encephalitis were considered. Early treatment allowed for the improvement of symptoms, although relapse could not be prevented.
